# Birth by caesarean section and semen quality in adulthood: a Danish population-based cohort study

**DOI:** 10.1186/s12978-024-01761-w

**Published:** 2024-03-08

**Authors:** Kun Huang, Anne Gaml-Sørensen, Nis Brix, Andreas Ernst, Linn Håkonsen Arendt, Jens Peter Ellekilde Bonde, Karin Sørig Hougaard, Gunnar Toft, Sandra Søgaard Tøttenborg, Cecilia Høst Ramlau-Hansen

**Affiliations:** 1 Department of Maternal, Child and Adolescent Health, School of Public Health, Anhui Medical University, No 81 Meishan Road, Hefei, 230032 China; 2 MOE Key Laboratory of Population Health Across Life Cycle, Hefei, 230032 China; 3 NHC Key Laboratory of Study On Abnormal Gametes and Reproductive Tract, Hefei, 230032 China; 4 Anhui Provincial Key Laboratory of Environment and Population Health Across the Life Course, Hefei, 230032 China; 5 Department of Public Health, Research Unit for Epidemiology, Aarhus University, 8000 Aarhus C, Denmark; 6 Department of Clinical Genetics, Aarhus University Hospital, 8200 Aarhus N, Denmark; 7 Department of Urology, Aarhus University Hospital, 8200 Aarhus N, Denmark; 8 Department of Obstetrics and Gynecology, Aarhus University Hospital, 8200 Aarhus N, Denmark; 9 Department of Occupational and Environmental Medicine, Bispebjerg and Frederiksberg Hospital, University of Copenhagen, 2400 Copenhagen, NV Denmark; 10 Department of Public Health, University of Copenhagen, 1353 Copenhagen K, Denmark; 11 National Research Centre for the Working Environment, 2100 Copenhagen, OE Denmark; 12 Steno Diabetes Center Aarhus, Aarhus University Hospital, 8200 Arhus N, Denmark

**Keywords:** Caesarean Section, Cohort Studies, Delivery, Obstetric, Fertility, Semen analysis

## Abstract

**Background:**

The caesarean section (CS) rate has increased worldwide and there is an increasing public and scientific interest in the potential long-term health consequences for the offspring. CS is related to persistent aberrant microbiota colonization in the offspring, which may negatively interfere with sex hormone homeostasis and thus potentially affect the reproductive health. It remains unknown whether adult sons’ semen quality is affected by CS. We hypothesize that CS is associated with lower semen quality.

**Methods:**

This study was based on the Fetal Programming of Semen Quality cohort (FEPOS, enrolled from 2017 to 2019) nested within the Danish National Birth Cohort (DNBC, enrolled from 1996 to 2002). A total of 5697 adult sons of mothers from the DNBC were invited to the FEPOS cohort, and 1044 young men participated in this study. Information on mode of delivery was extracted from the Danish Medical Birth Registry, and included vaginal delivery, elective CS before labor, emergency CS during labor and unspecified CS. The young men provided a semen sample for analysis of semen volume, sperm concentration, motility and morphology. Negative binomial regression models were applied to examine the association between CS and semen characteristics with estimation of relative differences in percentages with 95% confidence intervals (CIs).

**Results:**

Among included sons, 132 (13%) were born by CS. We found a slightly lower non-progressive sperm motility (reflecting higher progressive sperm motility) among sons born by CS compared to sons born by vaginal delivery [relative difference (95% CI): − 7.5% (− 14.1% to − 0.4%)]. No differences were observed for other sperm characteristics. When CS was further classified into elective CS, emergency CS and unspecified CS in a sensitivity analysis, no significant differences in non-progressive motility were observed among sons born by any of the three types of CS compared to sons born vaginally.

**Conclusions:**

This large population-based cohort study found no significant evidence for an adverse effect on semen quality in adult sons born by CS.

**Supplementary Information:**

The online version contains supplementary material available at 10.1186/s12978-024-01761-w.

## Plain language summary

Caesarean section is one of the most frequently used interventions during childbirth and global cesarean delivery rates continue to increase. The rising cesarean delivery rate has been reported to be related with series of adverse health outcomes in children, such as asthma, allergies, obesity, diabetes and even poor emotional, behavioral and educational outcomes. Still, it remains unknown whether children’s reproductive health is affected by this delivery mode.

Based on data from the Fetal Programming of Semen Quality cohort (FEPOS,) nested within the Danish National Birth Cohort, we have therefore analyzed the potential effect of caesarean section on son’s semen quality in 1044 young men. We found a slightly higher progressive sperm motility among sons born by caesarean section compared to sons born by vaginal delivery. No differences, however, were observed for semen volume, sperm concentration and morphology between the two delivery modes.

The FEPOS cohort is the largest population-based male offspring cohort worldwide. This is the first study aiming to examine the association between caesarean section and semen quality in adulthood. Although the findings need to be confirmed in other studies, it is reassuring that this large population-based cohort study finds no significant evidence for an adverse effect on semen quality in adult sons born by caesarean section.

**Supplementary Information:**

The online version contains supplementary material available at 10.1186/s12978-024-01761-w.

## Introduction

Worldwide, the caesarean section (CS) rate has almost doubled from 12% of all births in 2012 to 21% in 2015 [1] with large geographical differences. According to the World Health Organization, 6.2 million CSs are performed yearly without medical indication [2]. Understanding the long-term effects of CS on child health is important for guiding decision making for clinicians, policy makers and the parents. Potential consequences for the children’s respiratory, metabolic, cardiovascular and immune function affected by CS are described [3]. However, it remains unclear whether also the reproductive health of children is affected by CS.

There is scientific and public concern about the state of male reproductive health in Western countries [4]. Poor semen quality is a major cause of infertility [5], which affects psychosocial well-being of couples worldwide [6] and has major consequences for society, due to cost of treatment, absenteeism from work and, most importantly, lower birth rates [7, 8]. Whether mode of delivery impacts semen quality remains to be investigated. It has been indicated that infants born by CS may be colonized by aberrant microbiota [9–11] that may persist into adulthood [12]. Gut microbiota dysbiosis has been suggested to negatively modulate gonadal sex hormones production and regulation and thus potentially decrease reproductive fitness [13, 14]. In particular, it has been reported that gut microbiota in the infant born via elective CS was less rich and diverse than those born by emergency CS [15, 16], as may be due to “partial” microbial exposure during emergency CS following membrane rupture [15]. To the best of our knowledge, semen quality of sons delivered by CS has not previously been studied.

In this present population-based cohort study, we aimed to examine the potential association between birth by CS and the adult son’s semen quality, under the hypothesis that CS, in general, and elective CS, in particular, is associated with lower semen quality.

## Materials and methods

### Participants

This study is based on the Fetal Programming of Semen Quality (FEPOS) cohort [17], nested within the Danish National Birth Cohort (DNBC) [18]. In the DNBC, from 1996 to 2002, pregnant women were enrolled and interviewed by computer-assisted telephone interviews conducted around 16 and 30 weeks’ gestation, and at 6 months after childbirth.

The participants in the FEPOS cohort were recruited from March 2017 to December 2019. Recruitment to FEPOS is described in detail elsewhere [17]. In short, sons were eligible for invitation if their mothers responded to the first two telephone interviews during pregnancy in the DNBC and provided a gestational blood sample for the biobank within the DNBC. Invitation to the FEPOS cohort was further restricted to the following criteria: Son of at least 18 years and 9 months of age, living in close proximity to the FEPOS clinics in Copenhagen (the capital and largest city in Denmark) or Aarhus (the second largest city in Denmark). Sons were encouraged to decline participation if they had undergone sterilization, cancer treatment, orchidectomy, or had one or no testicles in the scrotum.

**Fig. 1:**
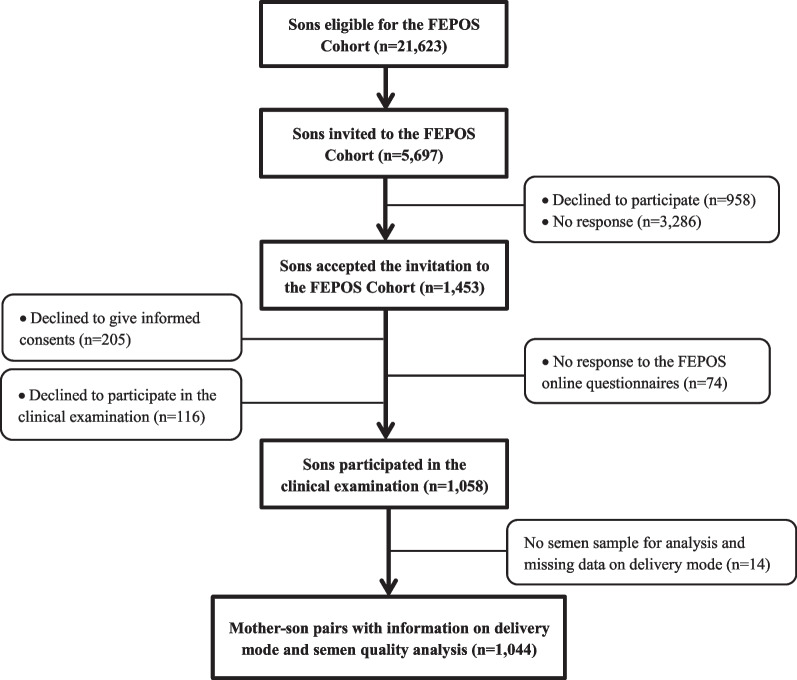
Flow chart of participants, the FEPOS Cohort, Denmark, 1998–2019

During the study period, 5697 of the 21,623 eligible young men were randomly invited to participate in FEPOS. Of these, 1174 (21%) responded to an online questionnaire on health behaviors, whereafter 1058 delivered a semen sample (participation rate 19%). After excluding sons with no semen sample and/or missing data on delivery mode, 1044 mother-son pairs (18%) were included in our main analysis (Fig. 1).

### Delivery mode

Information on delivery mode was extracted from the Danish Medical Birth Registry (DMBR) [19, 20] based on ICD-10 diagnostic codes and procedure codes according to the Nordic Medico-Statistical Committee classification of surgical procedures. Delivery mode included vaginal delivery (ICD-10: DO800, DO801, DO808, DO809, DO810, DO813, DO814, DO815, DO840, DO841), elective CS before labor (ICD-10: DO820 and operation codes KMCA10B or KMCA11), emergency CS during labor (ICD-10: DO821, DO842 and operation codes KMCA10D, KMCA10E) and unspecified CS (ICD-10: DO828, DO829 and operation codes KMCA10A). The latter three categories of CS (elective CS, emergency CS and unspecified CS) were merged into an overall CS exposure category in the main analysis due to low numbers in some of the separate categories.

### Semen quality characteristics

Participants provided a semen sample for analysis either at the clinic or at home. If collected at home, participants were recommended to keep the sample warm during transportation to the clinic and deliver the sample within one hour of ejaculation. Recommended abstinence time was 2–3 days.

Collection and analysis of semen followed recommendations by the WHO 2010 [21]. Analysis was conducted by two trained biomedical laboratory technicians and started within a maximum of two hours from ejaculation in 1029 out of 1044 semen samples (99%). In total, 786 (75%) semen samples were analyzed within one hour. Semen volume was measured by weighing of the sample in a pre-weighed container assuming 1 g = 1 ml. After liquefaction at 37 °C, sperm concentration was determined on two aliquots of semen using an Improved Neubauer Hemocytometer. Total sperm count was calculated by multiplication of semen volume and concentration. Sperm motility was determined by assessing progressive, non-progressive and immotile spermatozoa for at least 200 spermatozoa within each of two fresh drops of semen. The outcome used in the study was progressive motility, which, to ensure optimal model fit, was assessed as non-progressive motility modeled as non-progressive + immotile spermatozoa in percentage. Morphology was analyzed at the Reproductive Medicine Centre, Skåne University Hospital, in Malmö, Sweden. The percentage of normal spermatozoa was assessed in approximately 200 spermatozoa per slide.

External quality control with the European Society of Human Reproduction and Embryology (ESHRE) External Quality Assessment scheme (Centre for Andrology, Karolinska University Hospital, Stockholm, Sweden) indicated no distinct differences between the FEPOS laboratory technologists and the expert reference examiners [17].

### Covariates

Potential confounders were identified a priori using existing literature and Directed Acyclic Graphs (DAGs) [22] (Additional file 1: Appendix S1). Maternal age at delivery was retrieved from DMBR. Information on maternal smoking in the first trimester, pre-pregnancy body mass index (BMI) and highest socioeconomic status of the parents was provided by the mothers in the first DNBC interview. Socioeconomic status was defined according to the International Standard Class of Occupation and Education codes (ISCO-88 and ISCED). Categorization of the covariates was presented in Table 1.

**Table 1:** Baseline characteristics by delivery mode, N = 1044, the FEPOS cohort, Denmark, 1998–2019

Background characteristics	Delivery mode		Missing n (%)
	Vaginal delivery (n = 912)	CS (n = 132)	
Maternal characteristics			
Maternal age at delivery, years, mean ± SD	30.9 ± 0.1	31.4 ± 0.3	0 (0.0)
Highest socioeconomic status of parents, n (%)			0 (0.0)
High-grade professional	307 (33.7)	50 (37.9)	
Low-grade professional	307 (33.7)	40 (30.3)	
Skilled worker	171 (18.8)	25 (18.9)	
Unskilled worker	84 (9.2)	11 (8.3)	
Student, economically inactive and un-definable	43 (4.7)	6 (4.6)	
Pre-pregnancy BMI, kg/m^2^, n (%)			25 (2.4)
< 18.5	55 (6.2)	6 (4.7)	
18.5–24.9	663 (74.4)	85 (66.4)	
25.0–29.9	136 (15.3)	27 (21.1)	
≥ 30.0	37 (4.2)	10 (7.8)	
Smoking in the 1st trimester, n (%)			< 15 (< 1.4)^b^
Nonsmoker	702 (77.7)	101 (78.9)	
1–10 daily cigarettes	171 (18.9)	< 25 (< 18.9)^b^	
> 10 daily cigarettes	31 (3.4)	< 5 (< 3.8)^b^	
Pregnancy complications, n (%)^a^			0 (0.0)
None	860 (94.3)	120 (90.9)	
One or more	52 (5.7)	12 (9.1)	
Son’s characteristics			
Gestational week at delivery, mean ± SD	39.8 ± 1.5	39.3 ± 2.1	0 (0.0)
Birth weight, kg, mean ± SD	3.7 ± 0.5	3.6 ± 0.7	12 (1.1)
Semen-related characteristics			
Place of semen sample delivery, n (%)			6 (0.6)
At home	125 (13.7)	13 (9.9)	
In the clinic	781 (85.6)	119 (90.2)	
Abstinence time, days, mean ± SD	2.3 ± 0.0	2.4 ± 0.1	< 5 (< 0.5)^b^
Interval from ejaculation to analysis, minutes, mean ± SD	49.9 ± 0.7	50.2 ± 1.6	8 (0.8)
Spillage of semen sample, n (%)			5 (0.5)
No	747 (82.3)	112 (85.5)	
Yes	161 (17.7)	19 (14.5)	

*SD* standard deviation

Body mass index (BMI) = weight(kg)/height(m)^2^

^a^Pregnancy complications defined as women reporting one or more of the following conditions: diabetes mellitus (type 1, type 2 or gestational diabetes), hypertension and preeclampsia

^b^Due to data regulations that numbers less than five are not allowed to report, the numbers in the table have been changed or combined to cover up the numbers less than five

Information on precision variables was recorded at the clinical visit, including place of semen sample collection (at home or in the clinic), abstinence time (in days), spillage of semen sample (no; yes), and interval from ejaculation to analysis (in minutes).

Information on pregnancy complications, labor induction, gestational age at birth (in weeks) and birth weight (in grams) were used in sensitivity analyses. Pregnancy complications were self-reported in the women’s interview around 16 and 30 weeks of gestation and 6 months after childbirth and included diabetes mellitus (type 1, type 2 or gestational diabetes), hypertension and preeclampsia. Women, who reported one or several of these conditions, were defined as having pregnancy complications (none; one or more). Information on gestational age at birth, birth weight, and labor induction (pharmaceutical or non-pharmaceutical based on ICD diagnose codes DO837 and DO847 and procedure codes KMAC00, KMAC96A, BKHD20, BKHD20A and BKHD 21) were obtained from DMBR.

### Statistical analysis

Data management and statistical analyses were conducted with STATA MP, version 15.1 (StataCorp, College Station, TX). According to the requirements of regulations [GDPR, Regulation (EU), 2016/679 of 25 May 2018)], a calculated percentile must be based on at least five observations. Thus, pseudo percentile 5, 50 (median), and 95 for the semen characteristics were calculated by delivery mode using STATA’s -sumat- commands.

As the distributions of the semen characteristics were over-dispersed, we applied negative binomial regression models to examine the association between CS and semen characteristics using STATA’s -nbreg- package. We estimated crude and adjusted relative differences in percentages with 95% confidence intervals (CIs) for each semen characteristics by comparing sons born by CS to those born by vaginal delivery.

Azoospermic men were excluded from analyses of motility and morphology (n = 17), and participants reporting spillage of the semen sample were excluded from analyses of semen volume and total sperm count (n = 180). All models were adjusted for abovementioned selected confounders, place of semen sample collection and abstinence time. The analysis of sperm concentration and morphology was further adjusted for spillage, and the analysis of motility was further adjusted for interval from ejaculation as sperm motility decreases with time [23].

We applied selection weights to all analyses to account for non-participation [24]. These were estimated as the inverse probability of participation derived from a multivariable logistic regression with participation status (yes; no) as the dependent variable and the primary exposure variable (delivery mode) and the potential confounding factors, in addition to region (Aarhus; Copenhagen), parental time to pregnancy (TTP) including use of medically assisted reproduction (MAR), included as explanatory variables. TTP and MAR were considered indicators of parental fertility, and these data were obtained in the first interview in the DNBC.

Assumptions were checked for each model. We compared the observed distribution of each semen characteristic against the model-based distributions from the fitted model using QQ-plots. Furthermore, the standardized deviance residuals were plotted against the model-based predictions. The model check was compatible with the assumptions (data not shown).

We performed three sensitivity analyses to assess confounding by indication. First, we further adjusted for birth weight z-scores [25] as being small or large for gestational age might be an important indicator for CS, and birth weight for gestational age has been reported to be associated with semen quality [26, 27]. Birth weight z-score was not included in the main analyses due to the risk of collider-stratification bias [28, 29]. Second, we restricted our analyses to women without induction of labor and pregnancy complications. Labor induction has been suggested by some researchers as an independent risk factor for CS [30, 31], and the indications for induction may be the same as for CS. In addition, severe pregnancy complications might be a potential medical indicator for CS. Third, CS was further classified into elective CS, emergency CS and unspecified CS, with vaginally-born sons as reference, to examine whether the type of CS was important for semen quality.

## Results

Among the 1044 sons included, 132 (13%) were born by CS. Mothers giving birth by CS were on average older, had higher pre-pregnancy BMI and experienced more pregnancy complications than mothers giving birth vaginally (Table 1).

Overall, sons born by CS had similar median values for the assessed semen characteristics compared to sons born vaginally (Table 2).

**Table 2:** Semen characteristics ^a^ by delivery mode, the FEPOS cohort, Denmark, 1998–2019

Semen parameters	Number of observations	Vaginal delivery			Cesarean section		
		P_5_	Median	P_95_	P_5_	Median	P_95_
Semen volume, mL^b^	858	1.0	2.7	5.4	1.0	2.9	5.9
Total sperm count, million^b^	858	7.6	101.2	413.6	8.8	105.7	398.1
Sperm concentration, million/mL	1042	2.8	37.9	136.5	1.5	41.3	149.3
Progressive sperm motility, %^c^	1025	29.2	63.0	83.2	35.4	65.5	82.8
Morphologically normal sperm, %^c^	1020	0.5	6.0	15.0	0.1	6.0	14.5

^a^Pseudo percentiles were presented and were calculated based on the average of five values

^b^180 samples were excluded due to spillage

^c^Progressive motility and morphology were not available for 17 samples due to azoospermia

In the main analysis, we found a slightly lower percentage of non-progressive sperm motility (reflecting higher percentage of progressive sperm motility) for sons born by CS than for sons born vaginally [relative difference (95% CI): − 7.5% (− 14.1% to − 0.4%)]. No differences were observed for other sperm characteristics (Table 3).

**Table 3:** Relative differences in percentage in semen characteristics by delivery mode, the FEPOS cohort, Denmark, 1998–2019

Semen parameters	Number of observations^a^	Relative differences in percentage ^b^	
		Unadjusted model	Adjusted model
		%	% (95% CI)
Semen volume^f^	818	5.6 (− 6.8 to 18.5)	5.3 (− 5.3 to 17.1)^c^
Total sperm count^f^	818	0.9 (− 17.4 to 22.3)	− 0.8 (− 14.9 to 15.8)^c^
Sperm concentration	988	5.4 (− 11.7 to 24.2)	4.3 (− 11.2 to 22.5)^d^
Non-progressive motility^gh^	965	− 6.0 (− 13.8 to 1.3)	− 7.5 (− 14.1 to − 0.4)^e^
Morphologically normal sperm^g^	966	2.0 (− 11.5 to 17.4)	− 2.0 (− 14.2 to 11.8)^d^

^a^Number of observations in adjusted model for each parameters

^b^Relative percentage differences were obtained using sons born of vaginal delivery as the reference

^c^Adjusted for maternal age at delivery, highest socioeconomic status of parents, maternal pre-pregnancy BMI, maternal smoking in the 1st trimester, place of semen sample collection, abstinence time

^d^Further adjusted for spillage

^e^Further adjusted for spillage and interval from ejaculation to analysis

^f^180 samples were excluded for the analyses on semen volume and total sperm count due to spillage

^g^For non-progressive motility and morphology, 17 samples were not available for analyses due to azoospermia

^h^Non-progressive motility was modeled as (non-progressive + immotile spermatozoa) %, and a negative estimate indicated a relatively lower non-progressive motility (ie. higher progressive motility)

In the sensitivity analyses, with further adjustment for birth weight z-scores (model 1), or restriction to women without labor induction and pregnancy complications (model 2), the results did not change essentially. In model 3, when CS was further classified into elective CS, emergency CS and unspecified CS, no significant differences in non-progressive motility or any of the other semen characteristics were observed among sons born by any of the three types of CS compared to sons born vaginally (Table 4).

**Table 4:** Sensitive analyses for relative differences in percentage in semen characteristics by delivery mode, the FEPOS cohort, Denmark, 1998–2019

Semen parameters	Analysis strategies	Number of observations	Delivery mode	Relative differences in percentage	
				Unadjusted model	Adjusted model ^a^
				%	% (95% CI)
Semen volume^b^	Model 1^f^	806	Overall CS	5.6 (− 6.8 to 18.5)	5.4 (− 5.4 to 17.5)
	Model 2 g	691	Overall CS	5.5 (− 7.0 to 19.7)	5.2 (− 6.4 to 18.3)
	Model 3 h	818	Elective CS (n = 35)	− 4.7 (− 21.8 to 16.0)	− 2.4 (− 16.5 to 14.1)
			Emergency CS (n = 52)	10.9 (− 4.8 to 29.2)	10.1 (− 6.7 to 30.0)
			Unspecified CS (n = 17)	11.1 (− 14.5 to 44.5)	7.8 (− 9.9 to 29.0)
Total sperm count^b^	Model 1^f^	806	Overall CS	0.9 (− 17.4 to 22.3)	− 0.5 (− 14.8 to 16.1)
	Model 2 g	691	Overall CS	− 0.4 (− 19.7 to 23.5)	0.0 (− 15.9 to 19.0)
	Model 3 h	818	Elective CS (n = 35)	− 20.0 (− 41.7 to 9.7)	− 16.4 (− 36.7 to 10.5)
			Emergency CS (n = 52)	6.0 (− 18.5 to 37.8)	7.2 (− 12.0 to 31.0)
			Unspecified CS (n = 17)	29.4 (− 17.7 to 103.3)	9.5 (− 22.2 to 54.0)
Sperm concentration^c^	Model 1^f^	975	Overall CS	5.4 (− 11.7 to 24.2)	4.6 (− 11.2 to 23.3)
	Model 2 g	828	Overall CS	3.9 (− 13.4 to 24.4)	2.5 (− 14.0 to 22.3)
	Model 3 h	988	Elective CS (n = 41)	− 7.9 (− 29.1 to 21.0)	− 4.5 (− 27.6 to 26.0)
			Emergency CS (n = 60)	13.1 (− 9.6 to 41.3)	10.8 (− 11.3 to 38.5)
			Unspecified CS (n = 20)	8.7 (− 26.4 to 60.4)	4.1 (− 23.1 to 41.0)
Non− progressive motility^de^	Model 1^f^	952	Overall CS	− 6.0 (− 13.8 to 1.3)	− 7.3 (− 13.0 to 0.0)
	Model 2 g	812	Overall CS	− 5.4 (− 12.1 to 2.6)	− 6.8 (− 14.3 to 1.2)
	Model 3 h	965	Elective CS (n = 38)	− 9.0 (− 19.8 to 3.2)	− 10.8 (− 21.3 to 1.1)
			Emergency CS (n = 58)	− 6.7 (− 15.8 to 3.3)	− 7.4 (− 16.0 to 1.2)
			Unspecified CS (n = 20)	1.9 (− 14.6 to 21.3)	− 1.1 (− 16.8 to 17.9)
Morphologically normal sperm^ce^	Model 1^f^	953	Overall CS	2.0 (− 11.5 to 17.4)	− 2.1 (− 14.4 to 12.0)
	Model 2 g	812	Overall CS	1.6 (− 12.8 to 18.3)	− 2.0 (− 15.3 to 13.4)
	Model 3 h	966	Elective CS (n = 39)	− 19.4 (− 36.7 to 2.7)	− 20.4 (− 39.8 to 5.3)
			Emergency CS (n = 58)	11.4 (− 7.8 to 34.6)	5.6 (− 9.1 to 22.7)
			Unspecified CS (n = 20)	15.4 (− 16.5 to 59.4)	14.3 (− 11.9 to 48.4)

*CS* caesarean section

^a^Basic set of variables that were adjusted for all semen parameters included maternal age at delivery, highest socioeconomic status of parents, maternal pre-pregnancy BMI, maternal smoking in the 1st trimester, place of semen sample collection and abstinence time

^b^For the analyses of semen volume and total sperm count, 180 samples were excluded due to spillage

^c^For the analysis of sperm concentration and morphologically normal sperm, spillage was further adjusted for

^d^Non-progressive motility was modeled as (non-progressive + immotile spermatozoa) %, and a negative estimate indicated a relatively lower non-progressive motility (ie. higher progressive motility). In the analysis, spillage and internal from ejaculation to analysis were further adjusted for

^e^For non-progressive motility and morphology, 17 samples were not available for analyses due to azoospermia

^f^Model 1: Birth weight z-scores were further adjusted for

^g^Model 2: Restricted to women without labor induction and pregnancy complications

^h^Model 3: CS was further classified into elective CS, emergency CS and unspecified CS, and percentage differences were obtained by comparing with the sons born of vaginal delivery

## Discussion

To the best of our knowledge, this is the first study to examine the association between CS and semen quality in sons at adulthood. Overall, there was no significant evidence for an adverse effect of CS on semen quality.

There is no clear biological explanation for the slightly higher percentage of progressively motile spermatozoa among the young adults born by CS compared to sons born vaginally. Nevertheless, the effect size was small, and no associations were observed between CS and the other semen characteristics. Further, we did not observe associations when taking the different types of CS into account in sensitivity analysis. Therefore, the current result is likely a chance finding.

This study has several strengths. The FEPOS cohort is the largest population-based male offspring cohort worldwide with detailed and prospectively collected data on maternal health in pregnancy, including information on birth outcomes, which allowed for comprehensive confounding control and reduced the risk of recall bias. Besides the potential confounders, precision variables were adjusted for in the regression models to increase the statistical precision. As the prevalence of urogenital diseases was low in this population and are not suspected to affect the results, these diseases were not adjusted for in the analyses. Information on CS was prospectively registered, and data from the DMBR are considered valid [21] with high predictive values, although we cannot rule out some misclassification regarding the type of CS. Neither participants, nor the biomedical laboratory scientists conducting the semen quality analyses, were aware of the objective of the current study. Therefore, the risk of differential misclassification is limited.

Some limitations must be considered. The participation rate in FEPOS was 19%, which increases the risk of selection bias. However, we consider participation not to be related to CS (CS rate was 13% and 14% in participants and non-participants, respectively), and that self-selection into the cohort did not depend on semen quality. The sons were young and most would not yet be aware of their fertility status, which minimizes the risk of self-selection. However, sons from families with fertility problems could be more likely to participate. Therefore, we estimated selection weights, including parental time to pregnancy and MAR use, to take the potential selective participation into account. Further, in this study, we could not differentiate between indicators for elective CS. Medical indication for elective CS (e.g. preeclampsia) could interfere with the development of the male reproductive organs [32]. The DMBR have only registrations of elective CS on maternal request from late 2001 and onwards. We could therefore not differentiate between reasons for elective CS to examine if potential effects associated with CS could be attributed to the underlying medical conditions or the surgical procedure. We performed sensitivity analysis to assess confounding by indication by adjusting for birth weight for gestational age, excluding women with induction of labor and pregnancy complications and categorizing CS into elective, emergency and unspecified CS. Still, other sources of unmeasured confounding, in particular confounding by indication, cannot not be ruled out.

## Conclusions

In summary, this large population-based cohort study found no significant evidence for an adverse effect on semen quality in adult sons born by CS.

### Supplementary Information

**Additional file 1: Appendix S1.** DAG for CS and semen quality.

## Data Availability

The dataset analyzed in the study is not publicly available due to national data security legislation on sensitive personal data. Access to data can be applied for at dnbc-research@ssi.dk.

## Abbreviations

CS: Caesarean section
FEPOS: : Fetal Programming of Semen Quality
DNBC: : Danish National Birth Cohort
CI: : Confidence interval
OR: : Odds ratio
