# Supplementary figures and images for: Birth by caesarean section and semen quality in adulthood: a Danish population-based cohort study
Source: Reprod Health. 2024 Mar 8;21:33. doi: 10.1186/s12978-024-01761-w (PMC10921573; doi:10.1186/s12978-024-01761-w)

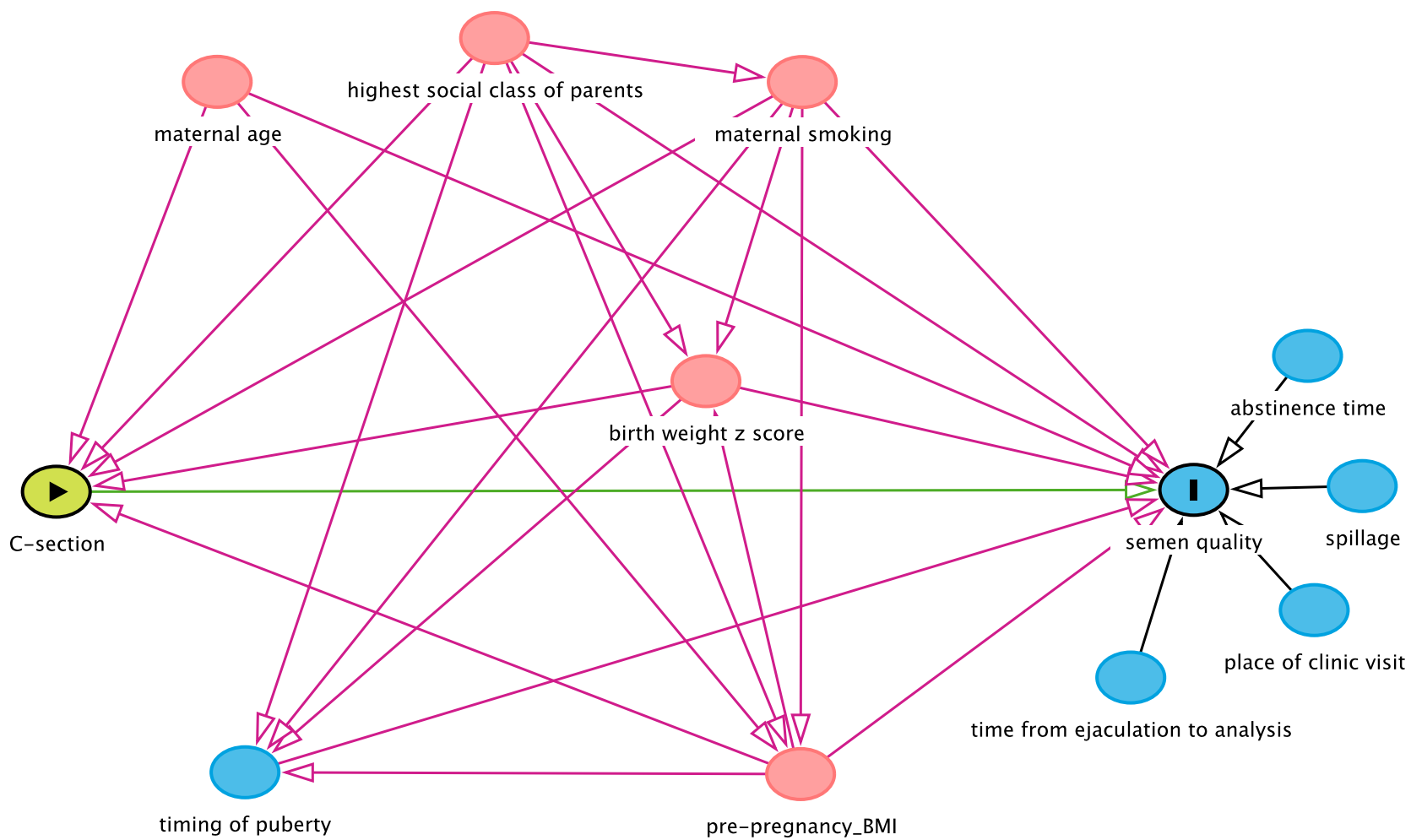

Supplement: Supplementary file 1 — Additional file 1: Appendix S1. DAG for CS and semen quality. [file 12978_2024_1761_MOESM1_ESM.pdf]
